# Engineering of autoactive NLRs: a big step toward breeding crops with durable and broad-spectrum resistance

**DOI:** 10.1007/s44307-025-00079-3

**Published:** 2025-08-20

**Authors:** Yan Wang, Shi Xiao

**Affiliations:** 1 MOE Key Laboratory of Bioinformatics, Center for Plant Biology, School of Life Science, Tsinghua University, Beijing, 100084 China; 2 Tsinghua-Peking Center for Life Sciences, Beijing, 100084 China; 3 State Key Laboratory of Biocontrol, Guangdong Provincial Key Laboratory of Plant Stress Biology, School of Agriculture and Biotechnology, Sun Yat-Sen University, Shenzhen, 518107 China

Plants rely on cell surface-localized pattern recognition receptors and intracellular nucleotide-binding leucine-rich repeat immune receptors (NLRs) to detect pathogen invasion and activate immune responses. NLRs specifically recognize effectors secreted by adapted pathogens, triggering a robust and durable defense known as effector-triggered immunity (ETI) (Cui et al. 2015). ETI is often characterized by rapid, localized cell death at infection sites, termed the hypersensitive response (HR). However, certain NLRs mediating antiviral immunity can confer extreme resistance without visible HR. Genetic studies have revealed that most plant disease resistance (*R*) genes encode NLR proteins, underscoring their pivotal role in immunity and their significant potential for breeding disease-resistant crops (Jones et al. 2024).

Traditional strategies for disease resistance breeding involve transferring of *R* genes into susceptible cultivars via conventional breeding or transgenesis. However, these approaches are time-consuming and often limited by the recipient's genetic background, resistance resources, or lack of durability due to rapid pathogen evolution (Mukhtar 2013; Zdrzalek et al. 2023; Zhao et al. 2024). Advances in understanding the structures and signaling mechanisms of immune receptors now enable knowledge-guided engineering of NLRs to acquire new or expanded recognition specificities. A recent breakthrough involving remodeling autoactive NLRs (aNLRs) offers a simple and effective strategy for engineering crops with broad-spectrum and durable disease resistance (Wang et al. 2025).

## Principles of aNLRs engineering

**Fig. 1:**
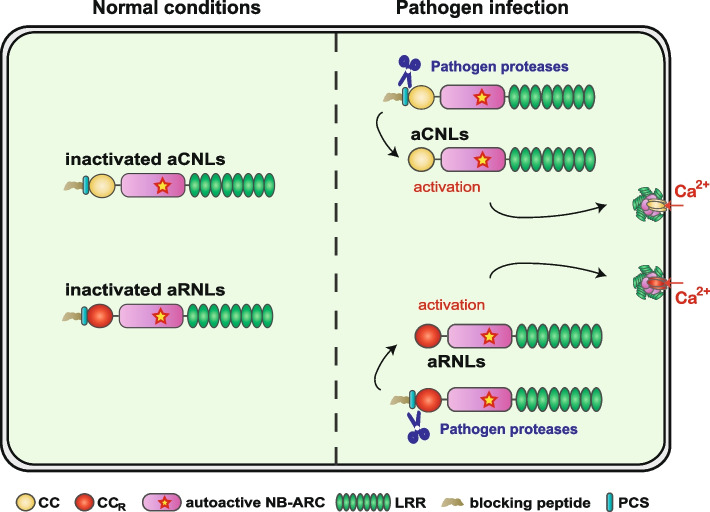
aNLRs engineering for broad-spectrum and durable disease resistance. aNLRs engineering is achieved by coupling an autoactive CNL or RNL protein with a pathogen-originated proteolytic cleavage site (PCS) and a blocking peptide at N-terminus. Under normal conditions, the autoactivity of aNLRs is blocked by the N-terminal peptide. Upon infection, protease secreted by pathogens cleave the PCS, remove the blocking peptide and release functional aNLR to trigger Ca^2+^ flux and a potent immune response

Plant NLRs are classified into three groups based on their variable N-terminal domains: Toll-like/interleukin-1 receptor resistance (TIR) domain-containing NLRs (TNLs), coiled-coil (CC) domain-containing NLRs (CNLs), and RESISTANCE TO POWDERY MILDEW 8-like CC (CC_R_) domain-containing NLRs (RNLs). Functionally, TNLs and CNLs serve as sensor NLRs that detect pathogen effectors, while RNLs act as helper NLRs that transduce sensor NLR signals into immune outputs (Huang et al. 2023). Cryo-EM and biochemical studies reveal that upon effector recognition, activated NLRs oligomerize into multi-protein complexes termed resistosomes. CNL and RNL resistosomes form Ca^2^⁺-permeable channels in the plasma membrane, triggering Ca^2^⁺ influx and ETI (Wang et al. 2023). Notably, an autoactive RNL, AtNRG1.1 (D485V), mimics NRG1 activation and oligomerization to form Ca^2+^-permeable channels (Jacob et al. 2021). Furthermore, the pore-forming activity of CNLs/RNLs depends on a short N-terminal helix within their CC/CC_R_ domains, and N-terminal fusions can block their ability to trigger immunity (Chen et al. 2017; Wang et al. 2019). Inspired by ETI induction by Ca^2+^-permeable resistosomes, Wang et al*.* have designed chimeric aNLRs comprising an autoactive CNL/RNL (aCNL/ aRNL) and an N-terminal blocking peptide coupled with a pathogen-originated protease cleavage site (PCS) (Fig. 1). The autoactivity of the aNLR is suppressed under normal conditions. However, upon infection, pathogen-encoded proteases cleave the PCS, remove the blocking peptide and precisely activate the aNLR to trigger a potent immune response (Wang et al. 2025).

## Application of aNLRs engineering in plants for broad-spectrum antiviral resistance

By incorporating PCS^PVY^ (YEVHHQ↓A), a target for cleavage by the NIa proteases encoded by more than 110 potyviruses, the authors have successfully engineered a autoactive forms of the CNL Tm-2^2^ (aTm-22) and the RNL AtNRG1.1 (a*At*NRG1.1) in transgenic tobacco plants. Transgenic tobacco plants expressing HA-PCS^PVY^-aTm-2^2^ or HA–PCS^PVY^–a*At*NRG1.1 confered complete or extreme resistance against multiple potyviruses. The resistance spectrum of engineered aAtNRG1.1 was further broadened by tandemly integrating PCS^PVY^ with PCS^TEV^, the PCS for NIa protease of tobacco etch potyvirus (TEV). This strategy was also successfully extended to soybeans. Transgenic soybeans expressing HA–PCS^SMV^–aAtNRG1.1 achieved complete resistance to soybean mosaic virus (SMV), a devastating global pathogen causing significant yield losses of soybeans.

## Advantages of aNLRs engineering over other strategies

Current NLR engineering strategies primarily focus on modifying sensor NLRs to alter effector recognition profiles. This is achieved through mutations or domain swapping in the leucine-rich repeat (LRR) domains or integrated decoy domains (IDs), which are responsible for direct effector binding. These approaches heavily depend on detailed structural knowledge of NLR-effector complexes. Expanding or creating new specificities using these strategies requires labor-intensive, site-directed modifications at binding interfaces, all while requiring careful avoidance of deleterious autoimmunity. An alternative strategy involves deploying the proteolytic PBS1 decoy system to broaden NLR specificity. It is reported that cleavage of PBS1 by the *Pseudomonas syringae* effector AvrPphB will induce activation of the guard NLR, RPS5. Substituting the native PCS within the PBS1 decoy protein with PCSs targeted by other pathogens enables RPS5 activation in response to infection of those pathogens and to initiate immune response (Kim et al., 2016). Nevertheless, this strategy is only effective in recipient plant possessing similar PBS1-guarding NLRs.

In contrast, aNLRs engineering offers several distinct advantages. First, its design is much simpler, requiring only a single autoactive RNL or CNL intolerant to N-terminal fusions and a short blocking peptide coupled with PCSs to control its activation. Second, aNLRs activation depends on cleavage of PCSs by the pathogen-encoded proteases, not specific effector binding. Using conserved PCSs (recognized by proteases from multiple related pathogens) or tandem PCSs readily enables broad-spectrum resistance. As proteases are ubiquitous among diverse pathogens and pests, this strategy holds potential for engineering resistance against virtually any pathogen/insect by simply exchanging the PCS within the chimeric aNLR. This dramatically expands the recognition scope of a single NLR, potentially even to cross-kingdom pathogens. Third, resistance engineered by this strategy is expected to be durable, provided that PCS-processing protease is essential for pathogen virulence/infection. Mutations in the protease that prevent PCS cleavage would simultaneously compromise pathogen fitness. Fourth, CNLs and RNLs are prevalent in crops, and their Ca^2^⁺-channel activity upon resistosome formation is a conserved mechanism. Unlike other NLR engineering approaches, which often depends on specific genetic backgrounds, aNLRs engineering should be broadly applicable across diverse crop species. Lastly, this strategy can be combined with CRISPR/Cas genome editing technology to remodel endogenous *CNL*/*RNL* genes directly and condition their autoactivity for plant immunity improvement. In summary, aNLRs engineering provides a simple yet powerful strategy for enhancing plant immunity, presenting significant opportunities for developing crops with durable, broad-spectrum disease resistance.
